# FGFR1 activation is an escape mechanism in human lung cancer cells resistant to afatinib, a pan-EGFR family kinase inhibitor

**DOI:** 10.18632/oncotarget.1866

**Published:** 2014-03-26

**Authors:** Koichi Azuma, Akihiko Kawahara, Kahori Sonoda, Kazutaka Nakashima, Kousuke Tashiro, Kosuke Watari, Hiroto Izumi, Masayoshi Kage, Michihiko Kuwano, Mayumi Ono, Tomoaki Hoshino

**Affiliations:** ^1^ Division of Respirology, Neurology, and Rheumatology, Department of Internal Medicine, Kurume University School of Medicine, Kurume, Fukuoka, Japan; ^2^ Department of Diagnostic Pathology, Kurume University Hospital, Kurume, Fukuoka, Japan; ^3^ Department of Pharmaceutical Oncology, Graduate School of Pharmaceutical Sciences, Kyushu University, Fukuoka, Japan; ^4^ Department of Bioscience and Biotechnology, Kyushu University, Fukuoka, Japan; ^5^ Department of Occupational Pneumology, Institute of Industrial Ecological Sciences, University of Occupational and Environmental Health, Kitakyushu, Japan; ^6^ Laboratory of Molecular Cancer Biology, Department of Clinical Pharmaceutics, Graduate School of Pharmaceutical Sciences, Kyushu University, Fukuoka, Japan

**Keywords:** activating EGFR mutation, FGFR1, non-small cell lung cancer, afatinib

## Abstract

Most NSCLC patients with *EGFR* mutations benefit from treatment with EGFR-TKIs, but the clinical efficacy of EGFR-TKIs is limited by the appearance of drug resistance. Multiple kinase inhibitors of EGFR family proteins such as afatinib have been newly developed to overcome such drug resistance. We established afatinib-resistant cell lines after chronic exposure of activating EGFR mutation-positive PC9 cells to afatinib. Afatinib-resistant cells showed following specific characteristics as compared to PC9: [1] Expression of EGFR family proteins and their phosphorylated molecules was markedly downregulated by selection of afatinib resistance; [2] Expression of FGFR1 and its ligand FGF2 was alternatively upregulated; [3] Treatment with anti-FGF2 neutralizing antibody blocked enhanced phosphorylation of FGFR in resistant clone; [4] Both resistant clones showed collateral sensitivity to PD173074, a small-molecule FGFR-TKIs, and treatment with either PD173074 or FGFR siRNA exacerbated suppression of both afatinib-resistant Akt and Erk phosphorylation when combined with afatinib; [5] Expression of twist was markedly augmented in resistant sublines, and twist knockdown specifically suppressed FGFR expression and cell survival. Together, enhanced expression of FGFR1 and FGF2 thus plays as an escape mechanism for cell survival of afatinib-resistant cancer cells, that may compensate the loss of EGFR-driven signaling pathway.

## INTRODUCTION

Lung cancer is the leading cause of cancer death worldwide (1). Somatic mutations in the epidermal growth factor receptor (EGFR) gene have been identified as a major determinant of the clinical efficacy of treatment with EGFR tyrosine kinase inhibitors (TKIs) such as gefitinib and erlotinib in patients with non-small cell lung cancer (NSCLC). Prospective clinical trials of EGFR-TKI treatment in NSCLC patients with *EGFR* mutations have demonstrated remarkable response rates of approximately 80% (2-8). Whereas most NSCLC patients with *EGFR* mutations benefit from treatment with EGFR-TKIs. However, almost all the individuals eventually develop resistance to these drugs.

Acquired resistance to EGFR-targeted drugs is one of the major obstacles to further improve clinical outcomes in this field. Further intensive research efforts have been focused on clarifying the mechanisms by which cancer cells acquire resistance to EGFR-targeted drugs (9, 10). T790M mutation, *Met* amplification, loss of PTEN, IGF-IR overexpression, and the AXL and Slug are reported to be the underlying mechanisms responsible for the EGFR-TKI resistance phenotype (11-16). The T790M mutation of *EGFR* has often been associated with acquired resistance to EGFR-TKIs in *EGFR* mutation-positive NSCLC. However, this mutation is present even in 31.5% of NSCLC patients pretreated with EGFR-TKIs, indicating that T790M is associated with de novo resistance (17, 18). Activation of alternative pathways, such as *Met* amplification or IGF-IR overexpression, has also been implicated in resistance to EGFR-TKIs in cells harboring activated *EGFR* mutation (12, 14). Furthermore, loss of PTEN and increased overexpression of MAPK, ABCG2, IGF1R, AXL, and BCL-2 have been reported as mechanisms of acquired resistance to EGFR-TKIs (9, 10). We have also reported that loss of PTEN expression and loss of activating EGFR gene allele results in acquisition of resistance to EGFR-TKIs in lung cancer cells harboring activated EGFR mutations (13, 19). However, the underlying mechanisms of resistance to EGFR-TKIs in patients with *EGFR* mutations have not been fully elucidated. The appearance of drug resistance in tumors during treatment of NSCLC patients with EGFR-TKIs has been a persistent obstacle.

In order to overcome drug resistance in relapsed NSCLC, multiple kinase-targeted drugs such as afatinib and ARQ197 have been further developed, and these are now being investigated in clinical trials (20, 21). Afatinib is an irreversible HER2/ErbB-family blocker that shows high affinity for EGFR T790M mutation. In phase III trials comparing afatinib with cisplatin and pemetrexed as first-line therapy, NSCLC patients with EGFR mutation had a higher response rate than patients without EGFR mutations when they received afatinib (22). In the present study, we invstigated how afatinib resistance was acquired in lung cancer cells, and also which oncogenic signaling pathway could be activated as a compensatory mechanism for cell survival. Here we report bypass activation of FGFR, and discuss the use of afatinib in combination with FGFR inhibitors for reversal strategy.

## RESULTS

### Establishment of afatinib-resistant lung cancer cells

**Figure 1:**
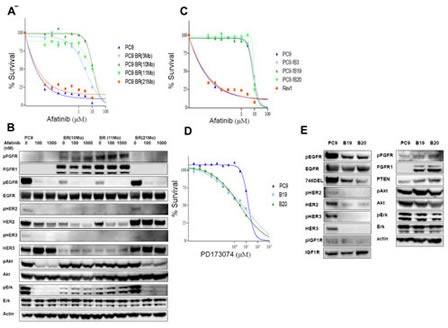
Establishment of afatinib-resistant lung cancer cells (A) Dose response curves for PC9, and drug-resistant PC9BR, PC9BR (3Mo), (10Mo), (11Mo), and (21Mo) cells to various doses of afatinib were determined by WST assay. (B) Western blotting analysis was performed for biochemical profiling of these cells in the absence or presence of afatinib for 6 h. Expression of pEGFR, HER2/pHER2, and HER3/pHER3 were markedly downregulated by resistance to afatinib, and activation of downstream regulating molecules for cell growth and survival was found to be highly resistant to the drugs. Downregulation of EGFR family proteins and upregulation of FGFR1 by selecting for afatinib resistance. (C) Dose response curves for afatinib were acquired for PC9 and its drug-resistant subclones, B3, B19, B20 and Rev1, with various doses of afatinib. (D) B19 and B20 showed 2- to 5-fold higher collateral sensitivity to PD173074.(E) Increasing expression of FGFR1 and pFGFR in resistant sublclones relative to their parental cells

The PC9 cells were grown initially in medium containing 0.01 μM afatinib, and the concentration of afatinib was gradually increased up to 1 μM over the following 11 months to establish the afatinib-resistant cell lines PC9 BR(3Mo), PC9BR(10Mo), and PC9BR(11Mo). We also established a revertant cell line, PC9 BR (21Mo), by culturing PC9 BR (11Mo) under drug free condition for 10 months. Dose response curves for PC9 and drug-resistant PC9 BR, PC9BR (3Mo), (10Mo), (11Mo) and (21Mo) cells to various doses of afatinib were determined by WST assay (Figure 1A). PC9BR (3Mo) cells that were selected after continuous exposure to the drug for 3 months already showed higher resistance, similar to that of PC9BR (10Mo) and PC9BR(11Mo). The IC_50_ values for each cell line were determined from the dose response curves for gefitinib and afatinib (Supplementary Table 1). PC9BR (3Mo), PC9BR (10Mo) and PC9BR (11Mo) cells were 3370-12900 times and 1170-135400 times more resistant to afatinib and gefinitib, respectively, than PC9 cells. By contrast, PC9BR (21Mo) cells showed similar sensitivity to both drugs as their parental PC9 cells (Supplementary Table 1), indicating that PC9 BR (21Mo) cells lost its drug resistant characteristic.

We then performed Western blotting analysis for biochemical profiling of these cells in the absence or presence of afatinib (Figure 1B). Drug-resistant PC9BR (10Mo) and PC9BR (11Mo) cells showed markedly decreased expression of pEGFR, HER2/pHER2, and HER3/pHER3 compared with PC9 and PC9BR (21Mo). By contrast, we observed increased expression of FGFR1 and pFGFR in the PC9BR (10Mo) and PC9BR (11Mo) cells relative to PC9 and PC9BR (21Mo) cells. Selection for afatinib resistance did not affect expression of EGFR expression. Phosphorylation of EGFR was susceptible to affatinib at 100 nM and 1000 nM in all of PC9 BR (10Mo), PC9BR (11Mo), PC9 and PC9BR (21Mo) cell lines. Afatinib markedly suppressed phosphorylation of Akt and Erk in PC9 and PC9BR (21M0) cells but not in PC9BR (10Mo) and PC9BR (11Mo) cells without affecting Akt and Erk expression (Figure 1B).

All of these cell lines did not harbor T790M mutation in the EGFR gene.

### Enhanced expression of FGFR1 by selection of afatinib resistance

To further characterize afatinib-resistant cells, we cloned three sublclones, PC9/B3 (B3), PC9/B19 (B19) and PC9/B20 (B20), from PC9BR (11Mo) cells, and Rev1 from PC9BR (21Mo) cells. Dose response curves for afatinib were obtained for PC9 and their three drug-resistant subclones in the presence of various doses of afatinib (Figure 1C). From the dose response curves, IC_50_ values were determined, and all resistant clones showed 750- to 880-fold higher resistance to afatinib than PC9(Table1). We also determined the dose response curves of PC9, B19 and B20 to various drugs (Supplementary Figure S1), and the IC_50_ values of these three cell lines for each drug were calculated(Table 1). Both afatinib-resistant sublclones showed more than 900-fold higher resistance to gefitinib, about 50-fold higher resistance to lapatinib, and 2-fold higher resistance to foretinib, respectively, than their parental PC9 cells. By contrast, B19 and B20 showed 2- to 5-fold higher collateral sensitivity to PD173074 (Figure 1D), an inhibitor of FGFR 1 and 3 tyrosine kinase (Table 1). The sensitivities of B19 and B20 cells to axitinib, dasatinib, cisplatin and paclitaxel were found to be similar to those of PC9 (Table 1).

**Table 1:** Comparison of sensitivity to various drugs between afatinib-resistant sublclones and their parental PC9 cells

		Relative drug resistance (IC50)		
Drugs	Targets	PC9	B19	B20
Afatinib	EGFR, HER2, HER3	1 (10 nM)	750 (7.5 μM)	880 (8.8 μM)
Gefitinib	EGFR	1 (10 nM)	2600 (260 μM)	960 (96 μM)
Lapatinib	EGFR, HER2	1 (0.27 μM)	52 (14.3 μM)	50 (13.5 μM)
Foretinib	Met	1 (0.7 μM)	2.7 (1.9 μM)	1.85 (1.3 μM)
PD173074	FGFR1,3	1 (15 μM)	0.5 (7.5 μM)	0.20 (2.9 μM)
Axitinib	PDGFR,VEGFR,	1 (2.4 μM)	1.3 (3.3 μM)	0.7 (1.7 μM)
Dasatinib	Src	1 (10 nM)	1 (10 μM)	1 (10 nM)
Cisplatin	DNA	1 (5.4 μM)	1.2 (6.9 μM)	1.5 (8.2 μM)
Paclitaxel	tubulin	1 (10 nM)	1 (10 nM)	1 (10 nM)

Therefore, we next compared expression levels of various growth factor receptors and their downstream regulatory molecules between PC9 and its resistant subclones (Figure 1D). Both resistant clones showed markedly decreased expression of pEGFR, and activated mutant EGFR (746del), HER2/pHER2, and HER3/pHER3 in comparison with PC9 cells. By contrast, there was no apparent change in the expression levels of IGF1R/p-IGF1R between the resistant subclones and PC9. We observed increased expression of FGFR1 and pFGFR in the resistant subclones relative to their parental counterpart (Figure 1E). Expression levels of unphosphorylated and phosphorylated Akt and Erk in PC9 and its drug-resistant subclones were similar.

Microarray analysis revealed that expression of FGFR2, FGFR3, and FGFR4 was only slightly or negligibly expressed in the resistant clones (unpublished data), suggesting that other FGFR family proteins except FGFR1 are unlikely to be involved in acquisition of drug resistance in B19 and B20 cells.

### Constitutive activation of FGFR through increased expression of both FGF2 and FGFR1 by acquisition of afatinib resistance

**Figure 2:**
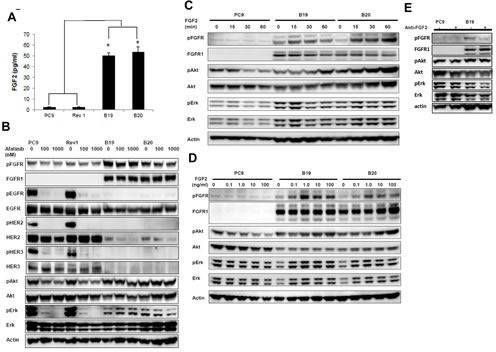
Increased expression of FGF2 and FGFR1 upon acquisition of afatinib resistance (A) Both resistant sublclones produced more than 30-fold higher levels of FGF2 than PC9 and Rev1. (B) Phosphorylation of EGFR, Akt and Erk in Rev1 was similarly susceptible to the inhibitory effect of afatinib (6 h) in PC9 when phosphorylation of Akt and Erk was resistant to the inhibitory effect of the drug in both resistant subclones. (C) Time kinetics for treatment with FGF showed enhanced phosphorylation of FGFR in both B19 and B20, accompanying by enhanced activation of Akt and Erk. (D) Increasing dose-dependent activation of FGFR, Akt and Erk in B19 and B20 upon treatment with various doses of FGF. All experiments were performed under serum free condition.(E) Autocrine stimulation of B19 by secreted endogenous growth factor was responsible for activation of FGFR phosphorylation

Since FGFR1 was constitutively phosphorylated in drug resistant clones, we examined whether FGFR was phosphorylated through an autocrine loop by its own FGF2 in resistant subclones. Using ELISA assay, we next compared the protein expression levels of FGF2 in serum-free conditioned medium among PC9, B19, B20, and Rev1 clones (Figure 2A). Both resistant sublclones produced more than 30-fold higher levels of FGF2 of about 50 pg/ml than PC9 and Rev1.

As shown in Figure 2B, we next compared the effect of afatinib on phosphorylation of EGFR family proteins, and their downstream signaling molecules and also the expression levels of FGFR1 among PC9, Rev1, and drug-resistant sublclones. Phosphorylation of EGFR, HER2 and HER3 was almost completely blocked in PC9, B19, B20, and Rev1 upon treatment with afatinib at 100 and 1000 nM (Figure 2B). By contrast, phosphorylation of Akt and Erk in both resistant subclones was not at all affected by afatinib. Expression of FGFR1 was also markedly upregulated in resistant sublclones relative to PC9, but its phosphorylation was not blocked by afatinib (Figure 2B). Furthermore, Rev1 showed similar expression levels of pEGFR to that of PC9, and EGFR phosphorylation was highly susceptible to afatinib as in PC9. Expression of FGFR1 was found to be markedly downregulated, as in PC9, and phosphorylation of Akt and Erk was also similarly susceptible to afatinib in Rev1. The restored sensitivity to afatinib in Rev1 was accompanied by both activation of EGFR and decreased activation of FGFR1.

We next compared the effect of exogenous addition of FGF2 on FGFR phosphorylation in PC9 and its drug-resistant subclones. Expression level of pFGFR were already higher in both resistant clones than in PC9 in the absence of FGF2. The time kinetics for treatment with FGF2 showed time-dependent enhancement of FGFR phosphorylation in both B19 and B20, accompanied by enhanced activation of Akt and Erk (Figure 2C). By contrast, no apparent phosphorylation of FGFR was observed in the parental PC9 cells. Figure 2D shows dose-dependent increased activation of FGFR and Akt and Erk in B19 and B20 when treated with various doses of FGF2. However, FGFR phosphorylation in PC9 was not augumented by FGF2. FGFR in both resistant sublclones thus seemed to be constitutively phosphorylated, and further phosphorylated in the presence of exogenous FGF2 (Figure 2C and 2D). We then investigated whether autocrine stimulation of B19 by secreted endogenous growth factor was responsible for activation of FGFR phosphorylation and was thus responsible for weaken of pFGFR and its downstream signaling (Figure 2E).

### FGFR activation is closely correlated with acquired resistance to afatinib

**Figure 3:**
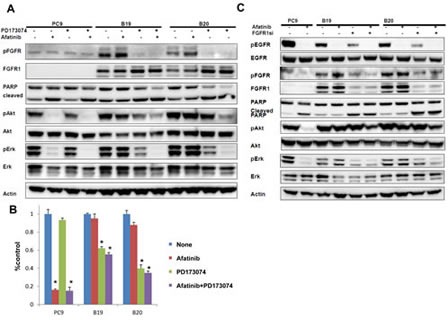
The close association of FGFR activation with acquired resistance to afatinib (A) Effect of FGFR-TKI against afatinib-resistant cells. The phosphorylation of FGFR was blocked upon treatment with either PD173074 (1 μM) alone or with both PD173074 (1 μM) and afatinib (1 μM) for 24 h. (B) Growth of both resistant sublclones was blocked upon treatent with PD173074 (1 μM) alone or with PD173074 (1 μM) and afatinib (1 μM). (C) Treatment with FGFR1 siRNA reduced the expression of FGFR1, accompanied by inhibition of both Akt and Erk phosphorylation in B19 and B20 cells. Cleaved PARP was also induced when resistant sublclones were treated with siRNA FGFR1 in the absence or presence of afatinib (1 μM) for 24 h.

We finally investigated whether FGFR was closely correlated with afatinib resistance in B19 and B20. Both resistant subclones were collaterally sensitive to an inhibitor of FGFR-TKI, PD173074 (Table1), and their FGFR was constitutively activated through an autocrine loop by FGF2. We first examined whether FGFR-TKI was able to block constitutive activation of Akt and Erk, which was not susceptible to the inhibitory effect of afatinib. The phosphorylation of FGFR was almost completely blocked by PD173074 alone and afatinib augmented this inhibitory effect in resistant subclones (Figure 3A). Apoptosis was also induced in two resistant clones by treatment with PD173074 alone or with both PD17074 and afatinib when assayed by PARP band cleavage.

We next examined whether the cell growth of drug-resistant clones was inhibited by FGFR-TKI. Cell growth of PC9 was blocked by afatinib alone, but not by PD173074 (Figure 3B). By contrast, there was marked growth inhibition of both resistant sublclones upon treatment with PD173074 alone or with both PD173074 and afatinib. We further examined whether FGFR1 knockdown by its cognate siRNA also exacerbated the inhibitory effect of afatinib on apoptosis and Akt/Erk phosphorylation in drug-resistant sublclones (Figure 3C). Silencing of FGFR1 reduced the expression of FGFR1, accompanied by inhibition of Erk phosphorylation but not Akt phospholylation in B19 and B20 cells (Figure 3C). Treatment with both of FGFR1-siRNA and afatinib further suppressed the phosphorylation of Akt and Erk. Cleaved PARP was also induced when resistant sublclones were treated with FGFR1 siRNA in the absence and presence of afatinib. Together, these findings suggest that the growth and survival of afatinib-resistant B19 and B20 cells become selectively addicted to the FGFR1 pathway during the selection of afatinib-resistant cells.

### Twist knockdown specifically blocked FGFR1 expression and Akt phospholylation in afatinib resistant cell lines

**Figure 4:**
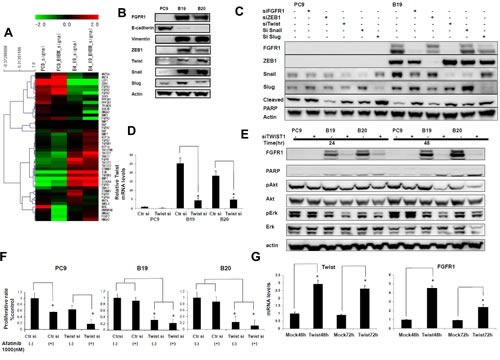
Twist knockdown specifically blocked FGFR1 expression and Akt phospholylation in afatinib resistant cell lines (A) Microarray analysis showed that the resistant subclones B19 acquired typical EMT characteristics relative to their drug-sensitive parental PC9. (B) Expression of Twist, Snail, Slug, and ZEB1 was increased in resistant cells, accompanied by a decrease in the expression of E-cadherin and an increase in that of vimentin. (C) Western blot analyses showed that expression of all three transcription factors was downregulated by their cognate siRNA. Phosphorylation of Akt and Erk was decreased when expression of twist was knockedowned. (D) Real-time PCR analysis revealed that expression of Twist mRNA was downregulated by its cognate siRNA by RT-PCR (E) Expression of FGFR1 was almost completely blocked accompanying by decreased phosphorylation of Akt and ERK when B19 or B20 cells were treated with Twist siRNA for 24hr and 48hr. (F) Cell growth inhibition of B19 and B20 when treated with afatinib and Twist siRNA. (G) FGFR1 mRNA levels in PC9cells were also increased to 2.5-4 folds of the control when twist was overexpressed.

We finally asked how FGFR1 expression was specifically augmented in resistant cells. Microarray analysis showed decreased expression of other FGFR family proteins, FGFR2, FGFR3 and FGFR4 in afatinib-resistant cell line when expression of FGFR1 was enhanced (Figure 4A). Figure 4A also showed increasing expression of Twist and Snail that are closely involved in transcription of EMT-related genes in resistant cells. Figure 4B also shows that expression of Twist, Snail, Slug, and ZEB1 was increased in resistant cells, accompanied by a decrease in the expression of E-cadherin and an increase in that of vimentin. We also observed morphological changes of fibroblast-line cell by selection of afatinib-resistant cells, accompanying by decreasing expression of E-cadherin with increasing expression of vimentin (data not shown).

We examined whether Snail and other related transcription factors were responsible for the enhanced expression of FGFR1 in drug resistant cell lines. Expression of ZEB1, Snail and Slug proteins was relatively much higher in B19 than PC9 (Figure 4C), and expression of Twist mRNA was also much higher in B19 and B20 than PC9 (Figure 4D). We confirmed that expression of Twist mRNA was downregulated by its cognate siRNA. Treatment with siRNAs for ZEB1, Twist, Snail and Slug resulted in markedly decreased expression of ZEB1, Snail and Slug proteins, and also Twist mRNA (Figure 4C and 4D).

As seen in Figure 4C, treatment with Twist siRNA, but not with ZEB1, Snail and Slug siRNAs, specifically suppressed expression of FGFR1 in resistant clones. Expression of FGFR1 was almost completely blocked, accompanying by decreased phosphorylation of Akt and ERK when B19 or B20 cells were treated with Twist siRNA for 24hr and 48hr (Figure 4E). We also observed cell growth inhibition of B19 and B20 when treated with Twist siRNA alone or with afatinib (Figure 4F). We next examined whether Twist overexpression might promote FGFR1 expression. FGFR1 mRNA levels in PC9 were found to be increased about 3 fold over the control when twist was overexpressed by transfection of Twist cDNA (Figure4G). Expression of FGFR1 thus seems to be specifically promoted by Twist than other transcription factors in afatinib-resistant clones.

## DISCUSSION

Our present study revealed novel characteristics of afatinib-resistant sublclones established from the drug-sensitive lung cancer cell line PC9 harboring the activated deletion E746-A750 mutant EGFR. In these afatinib-resistant sublclones, [1] expression of most of the EGFR protein family, including pEGFR, mutant EGFR, HER2 and HER3, and Met, was markedly downregulated; [2] they showed collateral sensitivity to PD173074 (FGFR-TKI); [3] there was alternatively enhanced expression of FGFR1 and its ligand FGF2, and phosphorylation of Akt and Erk was resistant to the inhibitory effect of afatinib; [4] of EMT-related transcriptional factors, Twist knockdown specifically reduced expression of FGFR1; and [5] afatinib together with either FGFR-TKI or FGFR1 knockdown markedly suppressed Akt and Erk phosphorylation, and cell growth and survival. Together, impaired expression of EGFR family proteins thus seems to compensatorily activate FGFR1-driven signaling pathway by acquired drug resistance to afatinib.

Acquisition of afatinib resistance resulted in markedly decreased expression of EGFR family proteins including activated EGFR, HER2 and HER3, which are targets for afatinib. This decreased expression of these EGFR family proteins might be mostly involved in acquisition of afatinib resistance. Our relevant study has recently demonstrated that loss of the activated mutant EGFR gene copy is closely associated with resistance to erlotinib and gefitinib, suggesting that expression levels of activated mutant EGFR can limit cellular sensitivity to such EGFR-TKIs (19). Furthermore, afatinib-resistant sublclones are also cross-resistant to gefitinib and also lapatinib (Table 1). EGFR forms a duplex with HER2 or HER3 (31), and sensitivity to lapatinib is controlled through HER2 and/or EGFR (32, 33). The cross-resistance to lapatinib in afatinib-resistant sublclones might be due to marked downregulation of HER2/pHER2 and pEGFR/activated mutant EGFR. With regard to the pleiotropic mechanisms involved in acquisition of resistance to EGFR-TKIs and other kinase inhibitors, the alternative pathway is one mechanism of escape from the cytotoxic or therapeutic effects of EGFR-targeted drugs (10). Activation of alternative pathways, such as *Met* amplification and IGF1R overexpression, has been implicated in resistance to EGFR-TKIs in non-small cell lung cancer cells bearing *EGFR* mutation (12, 14), and these molecules bypass the original oncogenic pathway to confer resistance to previously effective therapy. In afatinib-resistant sublclones, however, there was no altered expression of IGF1R (Figure 1D). and no phosphorylation of Met (date not shown), suggesting that the alternative pathway involving IGF1R and Met is unlikely to be involved in afatinib resistance.

**Figure 5:**
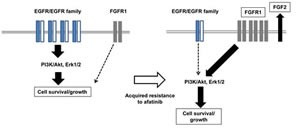
Our hypothetic model shows how afatinib resistance is acquired in lung cancer cells In drug sensitive cell line, the cell survival and growth of human lung cancer cells harboring activating EGFR depends upon the EGFR/EGFR family driven PI3K/Akt and Erk pathways, and this cell survival and growth is highly susceptible to afatinib and other EGFR-TKIs. By contrast, afatinib-resistant subclones express elevated levels of FGFR1 together with FGF2, resulting in activation of Akt and Erk, when EGFR/EGFR family-driven cell growth/survival signaling pathways are mostly attenuated. Of EMT-related transcription factors, Twist seems to specifically responsible for elevated expression of FGFR1 in afatinib resistant cell lines.

The FGFR tyrosine kinase family is consisted of 4 receptors and 23 ligands and activation of FGFRs is common oncogenic event (34). Recent study by Herrera-Abrea et al has demonstrated that EGFR limits drug sensitivity to FGFR tyrosine kinase inhibitor in FGFR3-mutant cell lines, and also that combination of FGFR and EGFR tyrosine kinase inhibitors overcome drug resistance to FGFR inhibitors, suggesting the close interaction of EGFR-and FGFR-driving cell growth or signaling pathways (35). In our present study using lung cancer cell lines, FGFR1 is most abundant receptor of the four family proteins in afatinib-resistant clones of PC9, and there was no enhancement in expression of other FGFR family proteins FGFR2, FGFR3 and FGFR4 (see Figure 4A). Ligand binding leads to FGFR1 dimerization, autophosphorylation, and activation of signaling components including Akt and Erk kinases, further affecting malignant transformation of cancer cells. We observed that the growth factor receptor-driven downstream molecules, Akt and Erk, were still highly phosphorylated in the presence of afatinib in resistant sublclones when expression of most of the EGFR family proteins was downregulated (Figure 1B, Figure 1E and Figure 2D). A possible mechanism underlying such activation of Akt and Erk in drug-resistant subclones treated with high doses of afatinib is that they induce increased expression of FGFR1 and pFGFR together with increased expression of FGF2 (see Figure5). Mark et al. have demonstrated various levels of expression of the FGF family proteins, FGFR1 and FGFR2, in NSCLC cell lines, and also shown that FGF2/FGFR1 autocrine signaling affects their sensitivities to gefitinib and FGFR-TKI (36). Both resistant sublclones, B19 and B20, showed more than 20-fold higher expression with 50 ng/ml FGF2 than their drug-sensitive counterpart cell lines, PC9 and Rev1 (Figure 2A). Both B19 and B20 already showed FGFR phosphorylation in the absence of exogenous FGF, suggesting an autocrine activation loop for FGF2-FGFR1 by afatinib resistance (Figure 2B and 2C). Exogenous addition of FGF further augmented FGFR phosphorylation and activation of both Akt and Erk in both resistant sublclones, but not at all in their parental counterpart PC9 cells (Figure 2B and 2C), suggesting the absence of FGF2-FGFR1 autocrine activation loop in PC9, possibly due to loss of active FGFR1 and FGF2 expression in the parental drug sensitive counterpart.

Concerning the possible link between FGF/FGFR and drug resistance to EGFR-TKIs, we have previously demonstrated amplification of the *FGFR2* gene in lapatinib-resistant breast cancer cells (37). Furthermore, Ware et al. (30) have reported that gefitinib-resistant cells after chronic exposure of several NSCLC cell lines to gefitinib showed increased expression of both mRNA and protein for FGFR1 and FGF2. A relevant study by Terai et al. has demonstrated that gefitinib-resistant sublclones from PC9 had enhanced expression of FGFR1 and FGF2, and also that gefitinib sensitivity in drug-resistant cells was restored by a combination of FGFR-TKI and gefitinib (38). Treatment with FGFR-TKI or FGFR knockdown also induced marked reduction of Akt and Erk activation in afatinib-resistant sublclones (Figure 3A and C). Co-adminstration of afatinib and FGFR-TKI also reduced apoptosis and suppression of cell growth in drug-resistant cells (Figure 3A, 3B, and 3C). These results strongly suggest that acquisition of afatinib resistance is due to oncogenic switch from activated EGFR family proteins to the FGF/FGFR signaling pathway (Figure5). FGFR1 may thus function as a survival factor for afatinib-resistant cancer cells, and activation of the FGFR-driven bypass signaling pathway confer resistance to previously effective therapy.

FGFR1 expression is often upregulated when epithelial cells are transformed into mesenchymal cells (39, 40). Microarray analysis demonstrated enhanced expression of EMT-related transcription factors such as Snail and Twist in afatinib-resistant clone (Figure 4A). Furthermore, we screened whether knockdown of these four EMT-related transcription factors could suppress phosphorylation of Akt and Erk in resistant clones in the presence of afatinib, and Twist knockdown specifically blocked Akt phosphorylation (unpublished data). Our present studies, clearly showed that increased expression of FGFR1 in afatinib-resistant clones was almost completely blocked only when treated with Twist siRNA (Figure 4B and 4D). Drug resistance to afatinib was also overcome by Twist knockdown in both resistant clone, B19 and B20, accompanying by suppression of both Akt and Erk activation (Figure 4D and E). Furthermore, transfection of Twist cDNA resulted in restored expression of FGFR1 in drug-resistant clone. It thus seems likely that Twist plays a pivotal role in enhanced expression of FGFR1 in resistant clones. Further study should be also required whether Twist alone plays a major role in overexpression of FGF2.

In conclusion, we have clarified one of the mechanism by which how PC9 cells acquired resistance to afatinib in vitro. Selection by afatinib resistance induced marked loss of the EGFR family proteins, EGFR, HER2 and HER3, together with inactivated EGFR family proteins, and simultaneously induced marked increases in the expression of FGF2 and activation of FGFR1. Such activation of the FGF/FGFR autocrine loop may have a compensatory role in promoting the survival and growth of afatinib-resistant cells. Whether this mechanism operates in patients with tumors refractory to EGFR-TKIs and multikinase inhibitors remains to be further studied.

## MATERIALS AND METHODS

### Cell culture and reagents

The human lung cancer cell line PC9 harboring del E746-A750 activating mutation in EGFR was maintained in RPMI1640 supplemented with 10% fetal bovine serum (FBS) and incubated in a humidified atmosphere of 5% CO_2_ at 37*°C*. The PC9 cells were kindly provided by Dr. Mayumi Ono (Kyushu University, Fukuoka, Japan) (13, 19, 23). Cells were routinely confirmed to be free of mycoplasma contamination using mycosensor QPCR Assay kits (Agilent Technologies). Afatinib, lapatinib, foretinib, gefitinib, and dasatinib were purchased from Selleck (Houston, USA). PD173074, cisplatin, paclitaxel and axitinib were from Sigma Aldrich (St. Louis, MO). The construction of pcDNA3-Twist has previously been described (24). The small interfering RNAs (siRNA) corresponding to FGFR1, Twist1, ZEB1, Snail, and Slug, mRNA and a non-specific siRNA (control) were purchased from Nippon Gene (Tokyo, Japan). Cells were transfected with siRNA duplexes using Lipofectamine RNAiMAX and Opti-MEM (Invitrogen, Carlsbad, CA) according to the manufacturer's recommendations.

### Western blot analysis

Western blot analysis was done as previously described (36) with antibodies for phosphorylated FGFR (pFGFR), FGFR1, p EGFR(Y1086), EGFR, pHER2(Y1221/1222), HER2, pHER3, HER3, pAkt, Akt, Erk, cleaved PARP, PARP, Vimentin, E-cadherin, Snail, Slug, ZEB1 (Cell Signaling Technology, Danvers, MA), Twist (Sigma,St. Louis, MO), and pERK (Santa Cruz Biotechnology, CA) or β-actin (Sigma,St. Louis, MO).

### Isolation of afatinib-resistant PC9 cells

To isolate afatinib-resistant cell lines, we cultured in increasing, step-wise doses of afatinib up to 1 μM over the following 11 months, and PC9 BR(3Mo), PC9BR(10Mo), and PC9BR(11Mo) were established (13, 19). We also established the revertant cells, PC9BR (21Mo), by culturing PC9BR (11Mo) cells under drug-free condition for 10 months and generated the subclones Rev1 from PC9BR (21Mo). Using limiting dilution, we further generated the clones B3, B19 and B20 from PC9 BR (11Mo). The identity of these clones was confirmed by analyzing their short tandem repeat profile using the Cell ID System (Promega, Madison, WI).

### Cell growth assay in vitro

Cells were plated in 96-well flat-bottomed plates and cultured for 24 h before exposure to various concentrations of drugs for 72 h. Cell counting kit 8 (WST-8 Doujindo, Kumamoto, Japan) was then added to each well, and the cells were incubated for 3 h at 37°C before measurement of absorbance at 450 nm with a Multilabel counter ARVO MX (PerkinElmer, USA). Absorbance values were expressed as a percentage of that for untreated cells, and the concentration of tested drugs resulting in 50% growth inhibition (IC_50_) was calculated using the Prism program (GraphPad, San Diego, CA). Triplicate wells were tested at each drug concentration.

### Quantitative real-time polymerase chain reaction and EGFR mutation analysis

Quantitative real-time PCR and EGFR mutation analysis was done as previously described (13, 25). All experiments were performed in a triplicate assays. To analyze the T790M mutation, exon 20 of the *EGFR* gene was amplified using the PCR primer set and TaKaRa Ex Taq polymerase (TaKaRa BIO, Inc). PCR products were directly used as templates for cycle sequencing reactions using the BigDye Terminator v1.1 Cycle Sequencing kit (Applied Biosystems). The forward or reverse primers were used for cycle sequencing reactions, which were carried out in an ABI PRISM 310 Genetic Analyzer.

### Gene expression microarrays

The cRNA was amplified, labeled, and hybridized to a 44K Agilent 60-mer oligomicroarray according to the manufacturer's instructions. All hybridized microarray slides were scanned by an Agilent scanner. Relative hybridization intensities and background hybridization values were calculated using the Agilent Feature Extraction Software program (9.5.1.1).

### Data analysis and filter criteria

Raw signal intensities and flags for each probe were calculated from the hybridization intensities, and spot information, according to the procedures recommended by Agilent. And the raw signal intensities of two samples were log2-transformed and normalized by a quantile algorithm (27) on the Bioconductor (28, 29). We selected probes that called the ‘P’ flag in both control and experimental samples. To identify up or down-regulated genes, we calculated Z-scores (29) and ratios (non-log scaled fold-change) from the normalized signal intensities of each probe. We thereafter established the criteria for the regulated genes: (up-regulated genes) Z-score ≥ 2.0 and ratio ≥ 1.5-fold, (down-regulated genes) Z-score ≤ −2.0 and ratio ≤ 0.66.

### Determination of FGF 2by ELISA

The concentrations of FGF2 in the conditioned medium were measured using commercially available ELISA kits (R&D Systems, Minneapolis, MN). Cells were plated in 24-well dishes in medium containing 10% FBS. When the cells reached subconfluence, the medium was replaced with RPMI1640 medium without FBS, and then the cells were incubated for a further 24 hours. The concentrations of FGF2 in the supernatants were measured using an ELISA kit in accordance with the manufacturer's protocols.

### Neutralizing FGF2 secretion

The autocrine role of FGF2 in cell proliferation was examined by adding an anti-FGF2 neutralizing monoclonal antibody (clone bFM-1: Millipore) at 5μg/ml for 12 hours. As a negative control, IgG was added.

### Statistical analysis

All tests were two-sided, and differences at *P* <0.05 were considered statistically significant. Statistical analysis was performed with JMP version 10 software (SAS Institute, Cary, NC).

## SUPPLEMENTARY TABLE AND FIGURE




