# Supplementary material for: FGFR1 activation is an escape mechanism in human lung cancer cells resistant to afatinib, a pan-EGFR family kinase inhibitor
Source: Oncotarget. 2014 Mar 26;5(15):5908–19. doi: 10.18632/oncotarget.1866 (PMC4171601; doi:10.18632/oncotarget.1866)
Supplement: Supplementary file 1 [file oncotarget-05-5908-s001.pdf]

Supplementary Table 1: Sensitivity of afatinib-resistant cells to afatinib and gefitinib.

Cell lines [a]	Relative drug resistance (IC50) [b]	
	Afatinib	Gefitinib
PC9	1 (10 nM)	1 (10 nM)
PC9 BR(3Mo)	3370 (3.3 μM)	1170 (11.7 μM)
PC9 BR (10Mo)	11600 (11.6 μM)	75200 (75.2 μM)
PC9 BR (11Mo)	12900 (12.9 μM)	135400 (135.4 μM)
PC9 BR (21Mo)	1 (10 nM)	1 (10 nM)

a) Drug sensitivity of PC9 and its afatinib-resistant derivatives selected after 3, 10, and 11 months of drug exposure. PC9BR (3Mo), PC9BR (10Mo), PC9BR (11Mo) and also PC9BR (21Mo), that were selected by continuous culture of PC9BR (11Mo) in the absence of the drug for 10 months, were examined.

b) IC_{50} values were calculated from the logistic regression lines obtained using triplicate assays, and resistance to each drug is expressed relative to the IC_{50} for PC9.
