# Supplementary figures and images for: FGFR1 activation is an escape mechanism in human lung cancer cells resistant to afatinib, a pan-EGFR family kinase inhibitor
Source: Oncotarget. 2014 Mar 26;5(15):5908–19. doi: 10.18632/oncotarget.1866 (PMC4171601; doi:10.18632/oncotarget.1866)

Fig supplement1

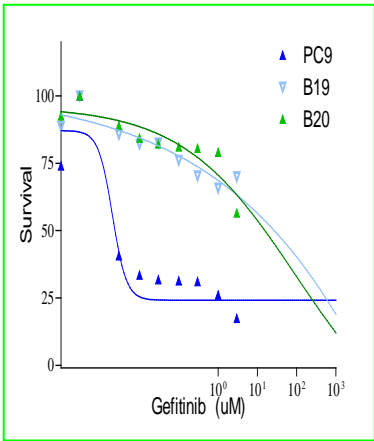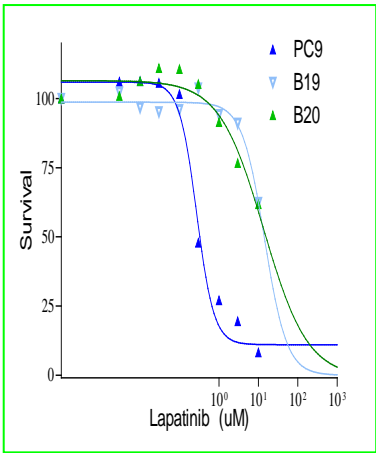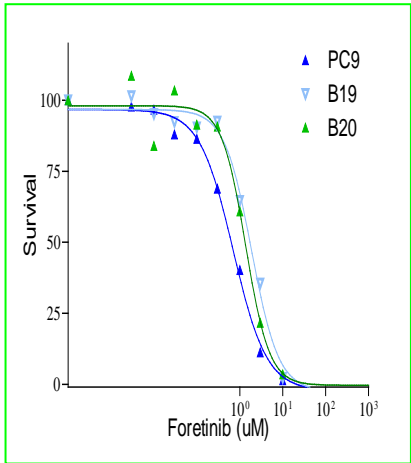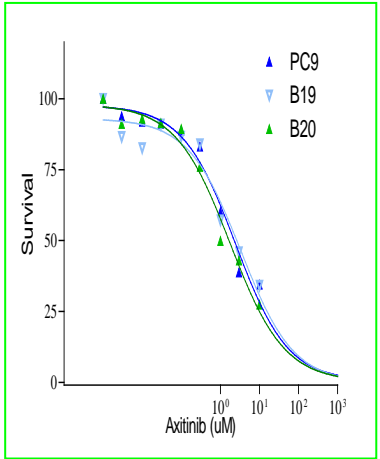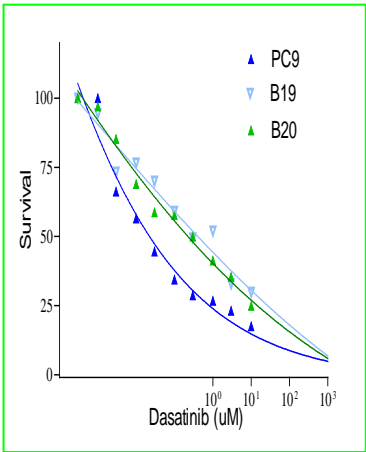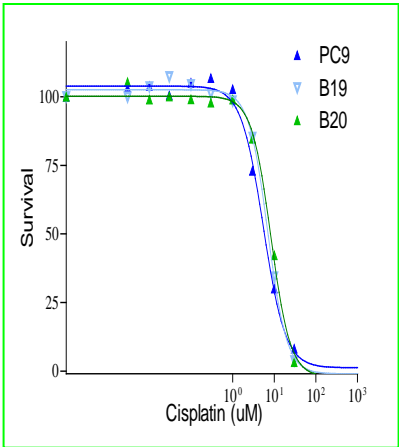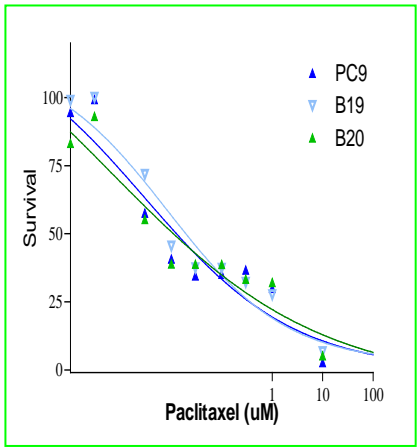

Supplement: Supplementary file 2 [file oncotarget-05-5908-s002.pdf]
